# Tailoring Interfacial Adhesion between PBAT Matrix and PTFE-Modified Microcrystalline Cellulose Additive for Advanced Composites

**DOI:** 10.3390/polym14101973

**Published:** 2022-05-12

**Authors:** Hongkun Wang, Xuran Liu, Jinfeng Liu, Min Wu, Yong Huang

**Affiliations:** 1 National Engineering Research Center of Engineering Plastics, Technical Institute of Physics and Chemistry, Chinese Academy of Sciences, Beijing 100190, China; wanghongkun18@mails.ucas.ac.cn (H.W.); liujinfeng17@mails.ucas.ac.cn (J.L.); 2 College of Materials Science and Opto-Electronic Technology, University of Chinese Academy of Sciences, Beijing 100049, China; 3 North China Institute of Aerospace Engineering, College of Material Engineering, Langfang 065000, China; liuxuran15@mails.ucas.ac.cn

**Keywords:** microcrystalline cellulose sheet, ball milling, surface energy, composite, interfacial adhesion strength

## Abstract

Cellulose materials have the potential to serve as sustainable reinforcement in polymer composites, but they suffer from challenges in improving interfacial compatibility with polymers through surface modification. Here, we propose adjusting the interfacial compatibility between microcrystalline cellulose (MCC) and poly (butylene adipate-co-terephthalate) (PBAT) through the strategy based on surface energy regulation. Mechanical ball milling with polytetrafluoroethylene (PTFE) powder was used to simultaneously pulverize, and surface modify MCC to produce MCC sheets with different surface energy. The modified MCC was used to reinforce PBAT composites by simple melt blending. The surface morphology, surface energy of MCC, and the amount of friction transferred PTFE during ball milling were characterized. The mechanical performance, composite morphology, crystallization behavior and dynamic thermomechanical analysis of the composites were investigated. The interfacial adhesion strength of composites closely relates to the surface energy of modified MCC. When the surface energy of MCC is closer to that of the PBAT matrix, it exhibits the better interfacial adhesion strength, resulting in the increased mechanical properties, crystallization temperature, storage modulus, and loss modulus. This work provides effective strategy for how to design fillers to obtain high-performance composites.

## 1. Introduction

Increased environmental awareness has boosted global interest in biodegradable and renewable materials, especially degradable polyesters, and a variety of bio-based nano reinforcing fillers, which helps reduce reliance on petroleum-based polymers to meet sustainability requirements [1,2]. Among natural degradable fillers, cellulose materials, including cellulose nanofibrils (CNF), cellulose nanocrystal (CNC) and microfibrillated cellulose (MFC), are promising bio-based filler. It not only has extraordinary transverse/axial modulus and mechanical strength, but also has good reactivity of surface hydroxyl groups and easy adjustment of surface properties, which are conducive to prepare high-performance polymer composites [3], especially for the composites of biodegradable aliphatic polyesters such as poly(β-hydroxybutyrate) (PHB), poly(ε-caprolactone) (PCL), poly (butylene adipate-co-terephthalate) (PBAT) and polylactic acid (PLA) [4,5].

The recent reported research on aliphatic polyesters filled with cellulose materials mainly focuses on the relationship between the hierarchical structure of cellulose and mechanical and thermal properties of polyester composites [6]. The unique characteristics of cellulose such as the surface polarity [7], dispersion degree [8], molecular orientation [9], the aspect ratio [10] and percolation degree of nanostructures [11] along with cellulose content [12] significantly affect the performance and macro-properties of polyester/cellulose composites. However, the most serious challenge is how to improve the interfacial incompatibility between hydrophilic cellulose and hydrophobic polyester [13]. One effective approach is reducing the hydrophilicity of cellulose, so as to optimize the compatibility with polymer matrix and improve the macro properties of polymer composites.

Chemical modifications have been employed to improve the hydrophobicity of cellulose to improve the compatibility between polyester and cellulose, which mainly includes surficial group conversion, small molecular substitution and polymer grafting [14]. As is well known, the mechanical properties of the composites are determined by the filler, polymer matrix, and the interface adhesion between the filler and the polymer. Thus, the mechanical behavior of polymer composites can be tuned by designing the matrix-filler interface adhesion strength [15]. The structure of the cellulose surface is conducive to improving the strength of interfacial adhesion between filler and polymer in the composite, especially in the case of the filler modified with grafted polymer and then mixed with the free chain of the same polymer [14]. In addition, the reduction of interfacial energy between cellulose and matrix is very important for the preparation of defect free composites. Grafting polymer onto cellulose surface, such as poly(lauryl methacrylate) (PLMA) [16], polylactic acid (PLA) [17,18], polycaprolactone (PCL) [19], poly(butylene succinate) (PBS) [20], quaternary ammonium cation surfactants [21,22] and cetyltrimethylammonium bromide (CTAB) [23], is one of the effective ways to reduce the polarity of cellulose and obtain the surface free energy equivalent to that of polyester matrix. Unfortunately, the process of polymer grafted-cellulose involves complex reactions and the extensive use of organic solvents, which limits the large-scale application. On the other hand, the surface wettability of lignocellulose can be controlled by changing lignin content, so as to customize the mechanical properties of composites. However, the regulation of lignin content shows limited change to the wettability of lignocellulose, the corresponding water contact angles were 35° and 78° for the lignin-free and the 14% lignin-containing nano paper [24,25]. Moreover, the content of lignin in lignocellulose depends on the delignification conditions. In addition, the dark color of lignin limits its application in composites [24].

Ball milling has gained widespread attention as a simple and efficient method of cellulose modification [26]. Huang, et al. developed a mechanochemical process to improve the hydrophobicity of cellulose surface by ball-milling in the presence of mixed acetic-oleic anhydrides used as esterifying agents [27,28]. In our previous works, the chemical modification of cellulose surface with dodecyl succinic anhydride was implemented during the ball milling process [29]. Then we found that cellulose milling in the presence of hydrophobic substances can form coated flat particles [30]. Furthermore, it was found that dry milling of cellulose in a polytetrafluoroethylene (PTFE) vessel leads to hydrophobization of cellulose surface and the achievement of water contact angle of 110–121° [31]. Similar results were obtained after cellulose milling in vessels made of high-density polyethylene (HDPE), polypropylene (PP), polyurethane (PU), and nylon. These results suggest that mechanochemical ball milling can be used for both esterification and polymer-coating of cellulose surfaces.

In this work, the solventless mechanical treatment of MCC and PTFE mixture was used to change the surface energy. It was found that ball milling caused the transfer of PTFE to MCC surface, resulting in the hydrophobic surface coating and continuously regulated hydrophobicity of the MCC particles. Then, MCC sheets, with different surface energies varying from 18.92 mJ/m^2^ to 64.27 mJ/m^2^, were used to fill PBAT by a melt blending technique as the target system for the study. Composites with different mechanical and thermal properties were obtained by changing the surface energy of MCC. This work provides a useful strategy on the performance design of green composites based on the cellulose-filled biodegradable polyesters.

## 2. Materials and Methods

### 2.1. Materials

PBAT was obtained from EcoWorld Co., Ltd., Hong Kong, China (FM0625, number average molecular weight: 54,000 g/mol, weight average molecular weight: 106,000 g/mol and PDI is 2). Microcrystalline cellulose was purchased from Linghuxinwang Chemical Co., Ltd. (Huzhou, China), with a degree of polymerization of about 200–220 and a molecular weight of about 36,000 g/mol. Poly(tetrafluoroethylene) micro powder was purchased from Innochem Co., Ltd. (Pyeongtaek, Korea) with particle size of 10–20 μm.

### 2.2. The Obtaining of Hydrophobic Coating on MCC Surface

MCC (60 g) and PTFE powder with an amount of 0 to 4 wt% were loaded into a 500 mL agate pot (Nanjing Nanda Instruments, Nanjing, China) containing 2 zirconia balls of 20 mm diameter, 100 zirconia balls of 10 mm diameter, and 300 zirconia balls of 6 mm diameter (631 g in total). Milling was carried out in a planetary mill QM-3SP4 (Nanjing Nanda Instruments) at 540 rpm for 3 h. The milling was done by repeated 60 min runs punctuated by 15 min pauses to avoid overheating. The obtained samples were labelled N#MCC, where N represents the percentage of PTFE addition.

### 2.3. The Preparation of PBAT/N#MCC Composites

N#MCC (2 wt%) and PBAT (98 wt%) particles was evenly mixed through a twin-screw extruder (Haake, Karlsruhe, Germany, with the screw diameter (D) of 11 mm and length of 440 mm (40 L/D)) to obtain the compounds. The screw speed was 50 rpm with a matching feeding speed of 5 rpm, and temperature profile from main feeder to die was applied as 170 °C, 170 °C, 180 °C, 180 °C, 180 °C, 180 °C, 170 °C and 170 °C. Standard test pieces were prepared from the obtained extruded compounds using an injection molding machine (MINIJET PRO, Haake, Karlsruhe, Germany) operating at an injection temperature of 180 °C and an injection pressure of 70 bar. The composite specimens were dumbbell-shaped with 75 mm (length) × 4 mm (width, narrow) × 2 mm (thickness) and labelled as PBAT/N#MCC.

### 2.4. Characterization

Scanning electron microscopy (SEM, Hitachi S-4800, Tokyo, Japan) was used to characterize the morphology of samples at an acceleration voltage of 10 kV. The N#MCC was dispersed in ethanol with a solid content of 0.05%, then 5 μL of which was spin-coated on the silicon wafer, followed by drying under fluorescent lamp before gold sputtering and observation. Then elemental mapping was carried out by HITACHI S-4800 equipped with an energy dispersive X-ray spectroscopy (EDS, Hitachi S-4800, Tokyo, Japan) The quenched spline of the PBAT/N#MCC composites were directly adhered to the conductive adhesive with 60 s gold sputtering and observed by SEM.

The surface topography of N#MCC was evaluated using the atomic force microscope (Bruker Multimode-8, Karlsruhe, Germany). The obtained images were analyzed with NanoScope Analysis software version 1.40 (Bruker Company, Karlsruhe, Germany). The powder sample was dispersed in ethanol and sonicated for 5 min in an ultrasonic cleaner (Kunshan Shu Mei KQ-250DE, Kunshan, China) to obtain good dispersion, then about 5 μL of the dispersion was deposited on a freshly cleaved mica plate and dried at room temperature.

The N#MCC powders were contracted under a 10 MPa pressure to obtain the sample plate with smooth surfaces. The contact angles of the N#MCC were measured on a contact angle meter (OCA20, Data Physics Instruments GmbH, Filderstadt, Germany) with 3 µL liquid drop at room temperature. The contact angles and surface free energies (γ) for two different probe liquids (water and DMF) were measured. We used the Owens-Wendt approach [32] to calculate the surface energy of N#MCC and analysis the dispersive and polar contributions in the surface energy according to Equation (1):

(1) $$\gamma_{L}\left(1+\cos\theta \right)=2\left(\sqrt{\gamma_{L}^{d}\gamma_{S}^{d}}+\sqrt{\gamma_{L}^{p}\gamma_{S}^{p}}\right)$$

where the *γ*, *γ^d^*, and *γ^p^* are the total dispersive, and polar surface energies, respectively. Meanwhile, the subscripts of “*L*” and “*S*” are the liquid drop and the solid surface, respectively. The *θ* is the contact angle between the solid substrate and liquid drops. The *γ_L_*, *γ^d^_L_* and *γ^p^_L_* of water are 72.8, 21.8 and 51.0 mJ/m^2^, respectively, while those of DMF are 37.3, 32.42, and 4.88 mJ/m^2^, respectively.

Furthermore, the interfacial energy can be calculated according to the harmonic mean Equation (2) or the geometric mean Equation (3) [33].

(2) $$\gamma_{12}=\gamma_{1}+\gamma_{2}-4\left(\frac{\gamma_{1}^{d}\gamma_{2}^{d}}{\gamma_{1}^{d}+\gamma_{2}^{d}}+\frac{\gamma_{1}^{p}\gamma_{2}^{p}}{\gamma_{1}^{p}+\gamma_{2}^{p}}\right)$$

(3) $$\gamma_{12}=\gamma_{1}+\gamma_{2}-2\left(\sqrt{\gamma_{1}^{d}\gamma_{2}^{d}}+\sqrt{\gamma_{1}^{p}\gamma_{2}^{p}}\right)$$

The interaction parameter *φ* proposed by Good-Girifalco can be used to characterize the two-phase polar surface energy matching relationship

(4) $$\varphi =\frac{W_{ad}}{{\left(W_{co1}\cdot W_{co2}\right)}^{1/2}}={\left(x_{1}^{d}x_{2}^{d}\right)}^{1/2}+{\left(x_{1}^{p}x_{2}^{p}\right)}^{1/2}$$

where *W_ad_* is the adhesion work between the two phases, *W_co_* is the cohesive work of the two phases, and *x^d^* = *γ^d^/γ, x^p^* = *γ^p^/γ*, *x^d^* + *x^p^* = 1. When the polarities of the two phases are the same, *φ* has a maximum value of 1, and as the polarity difference between the two phases increases, *φ* will decrease accordingly.

Mechanical properties of PBAT/N#MCC composites were measured by a universal mechanical testing machine (Intron5966, Boston, MA. USA) at a strain rate of 5 mm/min. The dumbbell-shaped spline size is 75 mm (length) × 4 mm (width) × 2 mm (thickness).

Differential scanning calorimetry (DSC, 1SET, Mettler, Zurich, Switzerland), thermal behavior was analyzed to investigate the crystallization ability of the composites.

During Non-isothermal crystallization, approximately 5 mg of neat PBAT, PBAT/N#MCC composites were heated from 20 °C to 200 °C at the heating rate of 10 °C/min under nitrogen purge, followed by a step of 200 °C for 5 min to eliminate thermal history. Then, the samples were cooled down to −50 °C at 20 °C min^−1^, maintained at −50 °C for 5 min and scanned at 10 °C/min up to 200 °C.

Glass transition temperature (T_g_), melt crystallization temperature (T_mc_) cold crystallization temperature (T_cc_), melting temperature (T_m_), cold crystallization enthalpy (∆H_cc_) and melting enthalpy (∆*H_m_*) were determined from the second heating scan. The crystallinity (*χ*) of neat PBAT, PBAT/N#MCC composites were calculated by:

(5) $$\chi_{c}=\frac{\Delta H_{m}}{\Delta H_{m}^{\theta }\times \left(1-fillers\;\mathrm{wt}\%\right)}\times 100\%$$

where *fillers* wt% is the weight fraction of N#MCC in the blend; $\Delta H_{m}$ is the enthalpy of melting; and $\Delta H_{m}^{\theta }$ is the enthalpy of melting of 100% pure PBAT taken as 114 J/g [34].

Dynamic mechanical analyzer (DMA/SDTA861e, TA. Instruments, New Castle, Delaware, USA) tests were adopted to perform compression molded samples with a thickness, width, and height of 1 mm, 4.5 mm, and 9 mm, respectively. The specimens were tested in the stretch mode at 20 mN, with a frequency of 1 Hz, and a heating rate of 3 °C/min in the range of −50 °C to 200 °C under nitrogen atmosphere.

## 3. Results and Discussion

### 3.1. Morphology of PTFE-Coated MCC

**Figure 1:**
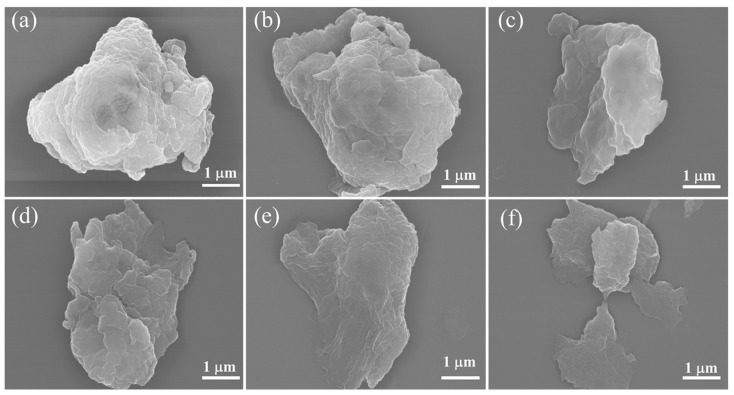
SEM images of ball milled microcrystalline cellulose with different PTFE addition, (**a**) 0 wt%, (**b**) 0.2 wt%, (**c**) 0.5 wt%, (**d**) 1 wt%, (**e**) 2 wt%, (**f**) 4 wt%.

Micro/nano MCC particles was prepared by a simple and efficient method of ball milling. The transfer mechanism of PTFE has been reported in our previous work [31]. The SEM images of the samples are shown in Figure 1 and Figure S2 (the raw date). The SEM image of the pristine MCC particle before the ball milling is shown in Figure S4. After 3 h ball milling, the diameter of MCC decreased from 75.6 μm to 2–5 μm. With the increase of PTFE addition, the particle size of cellulose decreased slightly, while the morphology of microcrystalline cellulose was gradually changed from the original granular to a flat shape. Moreover, element F was evenly distributed on the cellulose surface and became dense with the increase of PTFE addition (Figure S1).

**Figure 2:**
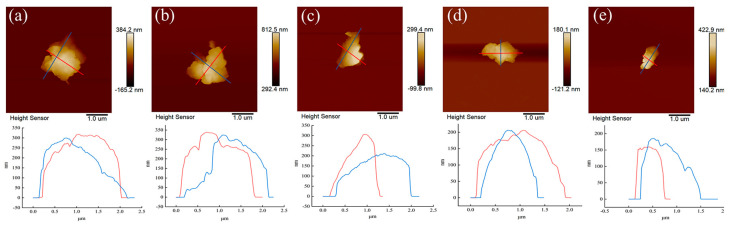
AFM images of ball milled microcrystalline cellulose with different PTFE addition, (**a**) 0.2 wt%, (**b**) 0.5 wt%, (**c**) 1 wt%, (**d**) 2 wt%, (**e**) 4 wt%.

To further study the thickness of microcrystalline cellulose sheets, the AFM images are shown in Figure 2. When the PTFE addition varied from 0.2–2 wt%, the thickness of the microcrystalline cellulose flakes gradually decreased from 350 nm to about 200 nm. However, with further increase of the PTFE addition, the thickness of the particles did not decrease significantly. In the process of ball milling, MCC particles and PTFE micro powder were subjected to pressure and shear force at the same time, resulting in instantaneous high temperature and high pressure [26,27,28]. As a result of friction and collision, MCC was broken into small particles and PTFE molecular chain was transferred to the cellulose surface. Thus, flat MCC particles coated with PTFE were formed. Due to the small friction coefficient of PTFE, which produced a lubricating effect and prevented the further transfer of excess PTFE to the cellulose surface, the thickness of the coated MCC particles did not decrease sharply when 4% PTFE was added.

### 3.2. Thermal Behavior of PTFE-Coated MCC

**Figure 3:**
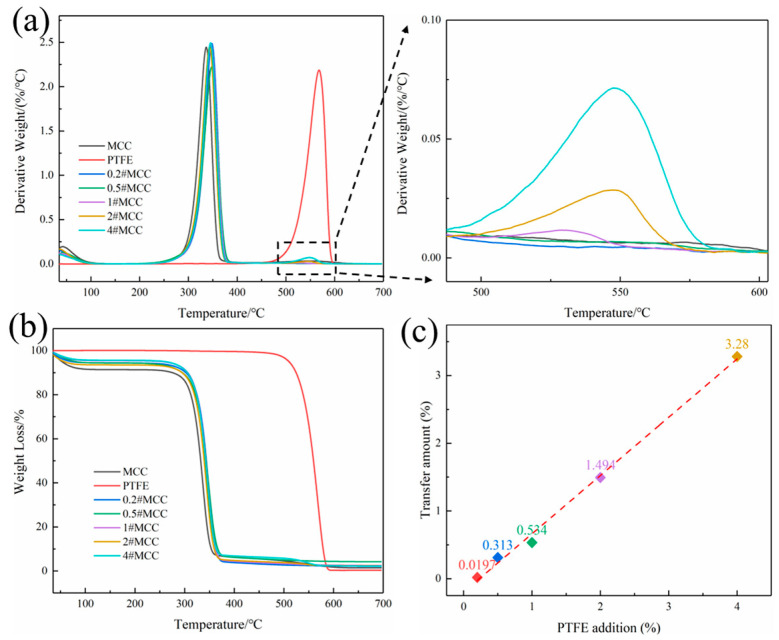
(**a**) DTG curves of PTFE and N#MCC samples; (**b**) TGA curves of PTFE and N#MCC samples; (**c**) Transfer amount of PTFE at different PTFE addition.

The transferred amount of PTFE was determined by thermogravimetric analysis (TGA), and the results are shown in Figure 3a,b. The thermal decomposition range of cellulose is different from that of PTFE. The main thermal decomposition temperature of cellulose is 240–395 °C, while that of PTFE is 520–600 °C. Therefore, the transfer amount of PTFE can be calculated through the weight loss of the two decomposition ranges. The dependence of friction transfer amount of PTFE on the total amount of PTFE additive to MCC during ball milling is shown in Figure 3c. The friction transfer amount of PTFE (y) increases almost linearly with the increase of the total addition amount of PFTE additive (x), and the fitting formula is *y* = 0.860*x* − 0.197. The negative intercept is attributed to the mass loss caused by some PTFE powder adhering to the zirconia balls and the inner wall of pot during ball milling. Besides the loss of PTFE, the linear increase of transfer amount indicates that the remaining PTFE were almost completely coated on the surface of MCC.

### 3.3. Wetting Properties of PTFE-Coated MCC

**Figure 4:**
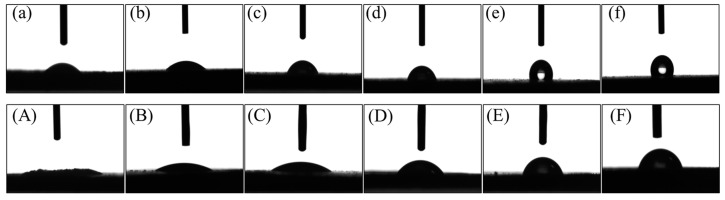
Water contact angle of (**a**) 0#MCC, (**b**) 0.2#MCC, (**c**) 0.5#MCC, (**d**) 1#MCC, (**e**) 2#MCC, (**f**) 4#MCC; DMF contact angle of (**A**) 0#MCC, (**B**) 0.2#MCC, (**C**) 0.5#MCC, (**D**) 1#MCC, (**E**) 2#MCC, (**F**) 4#MCC.

To investigate the coating of PTFE, the average contact angles of water and N,N-Dimethylformamide (DMF) on MCC and modified MCC were studied and the results are summarized in Table 1. Figure 4 shows the respective contact angles of water and DMF on pure MCC and PTFE coted MCC. Pure MCC with 3 h ball milling showed strong polarity, the contact angle of water and DMF were 34.2° and 8.8°, respectively, while the dispersive (van der Waals) contribution and the polar (acid-base) contribution were 12.08 mJ/m^2^ and 52.19 mJ/m^2^, respectively. With the increasing part of PTFE coating on the surface of MCC, the hydrophobicity of MCC increased, the contact angle of water increased from 38.6° (0.2#MCC) to 115° (4#MCC), and the surface energy decreased from 60.43 mJ/m^2^ to 18.92 mJ/m^2^, realizing the regulation of MCC surface from hydrophilic to hydrophobic. The results showed that the surface energy of MCC could be regulated through the simple mechanical ball milling in the presence of hydrophobic polymer. Compared with the hydrophobic modification methods such as grafting polymer and esterification on the surface of cellulose, the proposed method of cellulose hydrophobization has a great advantage since it is simple, highly efficient, and environmentally friendly.

### 3.4. Mechanical Properties of PBAT/N#MCC Composites

**Figure 5:**
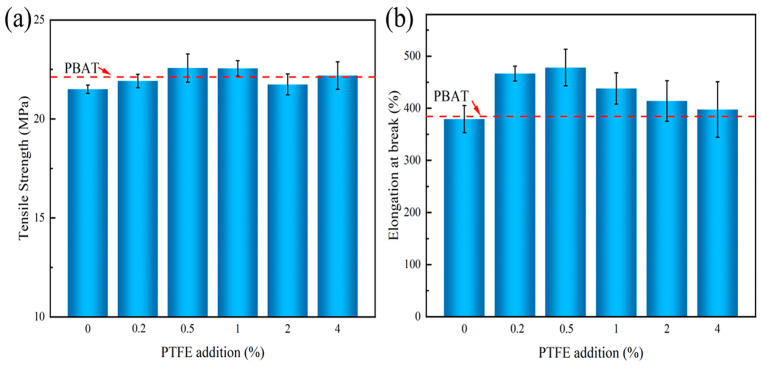
Mechanical properties of PBAT/N#MCC composites with different additions of PTFE (**a**) tensile strength, (**b**) elongation at break.

The mechanical properties of the PBAT/0.5#MCC composites with different 0.5#MCC additions was investigated, and the results showed that the optimal addition amount was 2% (Figure S5). The mechanical properties of PBAT composites having the filler content of 2% are shown in Figure 5. With the increase of coated PTFE addition, the tensile strength and elongation at break of the composites increased first and then decreased. As the reference line shown in Figure 5, the tensile strength of pure PBAT was 21.8 MPa and the elongation at break was 385.4%, respectively. When unmodified MCC was added as filler, the tensile strength and elongation at break of the composites decreased, especially the elongation at break decreased by 3.6%, indicating that hydrophilic MCC had poor compatibility with hydrophobic PBAT matrix [36]. Furthermore, compared with unmodified PBAT/0#MCC, the tensile strength and elongation at break of PBAT/0.5#MCC were increased by 5.1% and 28.8%. The results indicated that the difference of surface energy between filler and matrix was closely related to the change of mechanical properties. On the one hand, the mechanical properties are largely based on the effectiveness of stress transfer at the interface between filler and PBAT matrix; on the other hand, the quality of the interfacial bond depends on the wettability of the polymer and filler. Good infiltration makes the filler and polymer molecules in close contact and forms a strong intermolecular force, thereby improving the interfacial adhesion strength.

### 3.5. The Interface Adhesion between PBAT Matrix and PTFE-Modified MCC

**Figure 6:**
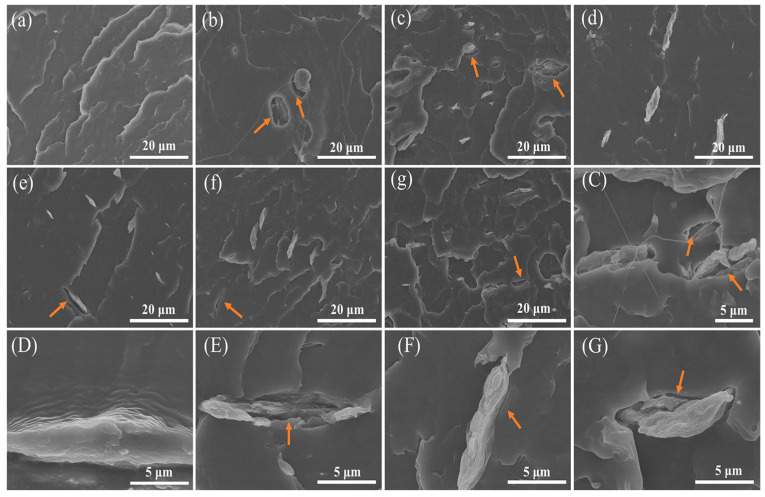
SEM fractography of composites, (**a**) pure PBAT, ×2k, (**b**) PBAT/0#MCC, ×2k, (**c**) PBAT/0.2#MCC, ×2k, (**d**) PBAT/0.5#MCC, ×2k, (**e**) PBAT/1#MCC, ×2k, (**f**) PBAT/2#MCC, ×2k, (**g**) PBAT/4#MCC, ×2k, (**C**) PBAT/0.2#MCC, ×5k, (**D**) PBAT/0.5#MCC, ×10k, (**E**) PBAT/1#MCC, ×8k, (**F**) PBAT/2#MCC, ×8k, (**G**) PBAT/4#MCC, ×10k.

The interface compatibility between modified MCC and PBAT was observed, and the results are shown in Figure 6 and Figure S3 (the raw date). The fracture section of pure PBAT was flat and clean, while the addition of unmodified MCC lead to obvious voids and holes pulled out by particles, indicating that the interface between unmodified MCC particles and matrix was poor. As shown in Figure 6c–g, different amount of PTFE coated MCC were evenly distributed in the PBAT matrix, but the holes left by the pulling out of sheets and the gap with the matrix can be seen obviously, as indicated by the yellow arrow. Further increase in magnification of the interface images (Figure 6C–G). There were different degrees of gaps between different amount of PTFE coated MCC and the matrix, but it is worth noting that 0.5#MCC was closely combined with PBAT matrix, and no obvious gap at the interface was observed. As a result, it can be concluded that the interfacial adhesion strength between modified MCC and PBAT matrix was the main factor affecting the mechanical properties of the composites.

**Figure 7:**
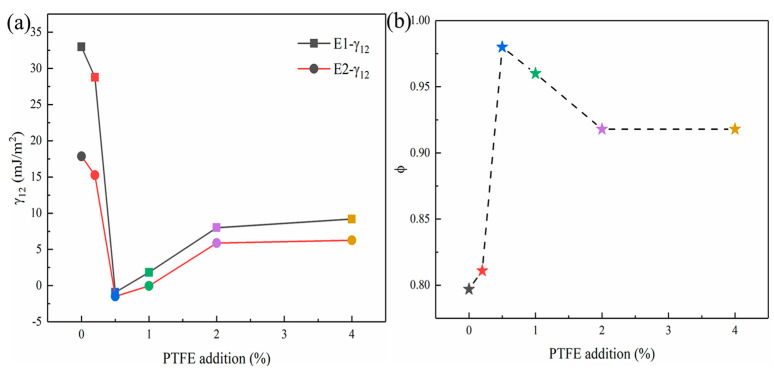
(**a**) the interfacial energy between modified MCC with different PTFE additions and PBAT calculated by two different formulas; (**b**) interaction parameter.

For the same polymer matrix, the change of surface free energy of filler will cause the change of interface energy and adhesion work. the interfacial energy E1-γ_12_ and E2-γ_12_ were calculated according to the harmonic mean Equation (2) and the geometric mean Equation (3), and Equations (2)–(4) was used to calculate the interfacial interaction parameters respectively, the results were showed in Figure 7a,b and Table 2. The interface energy represents the free energy required for the combination of two different interfaces. The smaller the interface energy, the less the free energy provided by the outside is required for the interface combination between the two phases. With the increase of PTFE addition, the interface energy between modified MCC and PBAT first decreased and then increased to become stable. The minimum value appeared when the PTFE addition was 0.5%, and the trend of the results calculated by the two formulas was consistent (Figure 7a). In addition, the higher the adhesion work, the more work required to separate the material and the better the mechanical properties of the material. Here, the interaction parameter *φ* was used to represent the magnitude of the adhesion work, and the closer the *φ* value was to 1, indicating that the higher the two-phase adhesion work and the greater the interfacial adhesion strength. As shown in Figure 7b, with the increase of PTFE addition, the interaction parameters between modified MCC and PBAT first increased and then decreased to a stable level. When the PTFE addition was 0.5%, the maximum value of *φ* was 0.98, which was very close to 1, indicating that 0.5#MCC and PBAT had very high interfacial adhesion strength, which was consistent with the results observed by SEM and mechanical performance. Thus, it can be concluded that strong cellulose/polymer interaction is conducive to stress transfer and enhance mechanical properties [37].

In addition, the polar interaction *γ^p^* has great influence on the binding mechanism. When two solid materials contact each other (at the molecular or atomic level), two polar components and two dispersive components interact respectively. However, if there is a non-polar phase in the contact object (such as polytetrafluoroethylene), only the dispersive component interacts, and the bonding strength between them is much weaker than that under the same conditions [38]. Therefore, with the further increase of PTFE transfer amount (such as 2#MCC and 4#MCC), the dispersion component accounts were the main part and the polar component accounts were a small proportion. Therefore, the interface adhesions between 2#MCC and 4#MCC with PBAT matrix were weakened, the interaction parameter *φ* decreased, and the macroscopic performance was the decline of the mechanical properties of the composite.

### 3.6. Thermal Behavior of PBAT/N#MCC Composites

**Figure 8:**
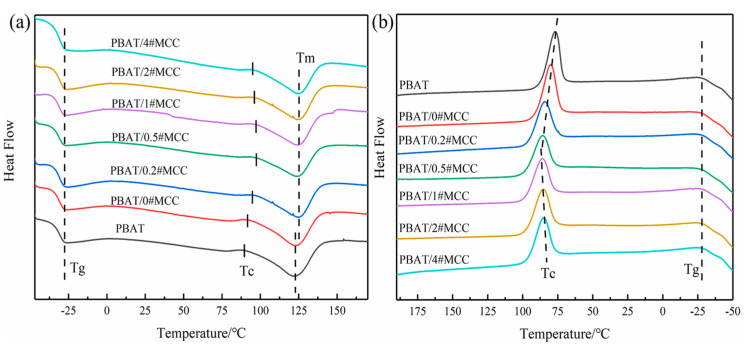
DSC results for PBAT/N#MCC composites, (**a**) the second heating scan, (**b**) the second cooling scan.

It is well known that the surface properties of fillers will affect the thermal movement of polymer segments [39]. Non-isothermal crystallization tests were carried out on the composites, and the results are shown in Figure 8. The results showed that the addition of PTFE coated MCC made little change of T_g_. The T_g_ of PBAT was low (about −30 °C) and the movement ability of chain segment was limited. Therefore, the change of the surface energies of modified MCC did not significantly change the glass transition temperature of PBAT composite. The effect of fillers on the movement of polymer segments was investigated by the change of crystallization temperature. According to the principle of polymer crystallization, the difficulty of polymer crystallization in the process of temperature change can be evaluated by ∆T, which was the difference between the melting endothermic peak temperature T_m_ at constant temperature rise and the crystallization exothermic peak temperature T_c_ at constant temperature drop [40]. The ∆T values are shown in Table 3, it can be seen that the addition of PTFE coated MCC significantly reduced ∆T, suggesting that the melt tended to form crystal nucleus and promoted crystallization when cooling down. However, the crystallinity of the composite was not improved compared with pure PBAT. Moreover, the XRD results also showed that there was no shift of diffraction peaks happened for the PBAT/N#MCC composites compared to pure PBAT, which indicated that the crystal structure of PBAT did not change with the addition of PTFE coated MCC, but the apparent crystal size of PBAT decreased slightly (Figure S6 and Table S2). On the one hand, the interfacial adhesion led to the formation of interfacial layer, which was not conducive to heterogeneous nucleation effect. On the other hand, the reduction of interfacial energy was conducive to the movement and arrangement of polymer chain segments along the filler surface, and the rearrangement of molecular chains into the lattice became easier, which was manifested by the increase of crystallization temperature. Therefore, the decrease of interfacial energy and the increase of interfacial adhesion strength led to the increase of crystallization temperature but the decrease of crystallinity and apparent crystal size of PBAT.

The results of non-isothermal crystallization test show that there is a complex relationship between interfacial compatibility and crystallinity, while the relationship between crystallization temperature and interfacial energy is relatively clear. The lower the interfacial energy between the two phases, the easier it is for the molecular chains to rearrange into the lattice, and the higher the crystallization temperature [41]. This is consistent with the findings of Hu et al. [42], when a small amount of PET-m-phthalic acid-5-sulfonic acid sodium copolymer was added to PET/aromatic polyamide system as compatibilizer, the T_hc_ of PET gradually increased with the increase of compatibilizer content. It is also consistent with the results of Huang et al. [28], which used mechanical ball milling method and acetic acid oleic acid mixed anhydride as esterification agent to improve the surface hydrophobicity of cellulose. The T_c_ of PP composite increased with the increase of cellulose substitution degree (DSa values of the 30 BMCP and 240 BMCP were 0.43 and 1.01, respectively), and the macro mechanical properties also increased, but the change of crystallinity was not obvious.

### 3.7. Viscoelastic Properties of PBAT/N#MCC Composites

**Figure 9:**
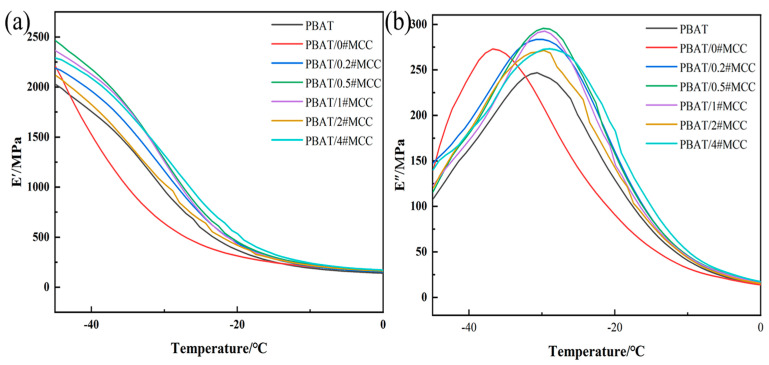
DMA results for PBAT/N#MCC composites, (**a**) storage modulus, (**b**) loss modulus.

Viscoelastic properties of PBAT/N#MCC composite were investigated using dynamic mechanical analyzer. The results are shown in Figure 9 and Table S1. Before the glass transition temperature (−30 °C), the addition of unmodified MCC resulted in a decrease in the storage modulus of the composites compared to pure PBAT, an increase in the loss modulus, and a shift in the T_g_ value of the composites to lower temperatures, indicating that the MCC particles with high surface energy had weak interface bonding strength with the PBAT matrix. There was a large gap between the granular MCC and the matrix (Figure 6b), the chains were less bound and the free volume of movement increases, resulting in a decrease in the storage modulus and a decrease in the glass transition temperature. When PTFE coated MCC was added, all samples exhibited higher storage and loss modulus than the PBAT matrix at −40 °C, indicating that the interaction between filler and matrix segments was enhanced. It was worth noting that at −40 °C, the storage modulus of PBAT/0.5#MCC was 42.9% and 24.3% higher than that of PBAT/0#MCC and PBAT, respectively, indicating that the interface interaction between 0.5#MCC and PBAT matrix was strong, and the increase of interface adhesion work resulted in the increased storage modulus and loss modulus.

## 4. Conclusions

In this work, a facile mechanical ball milling method was adapted to prepare PTFE-coated MCC and surface energy of modified MCC was tuned by adding different amount of PTFE. The MCC particles, with surface energies varying from 18.92 mJ/m^2^ to 64.27 mJ/m^2^, were particles with typical dimensions of 2–5 μm wide and 100–400 nm thick. It was found that the mechanical and thermal properties of the composites were closely related to the interface energy and adhesion work between the two phases. The properties of PBAT composites can be controlled by designing the surface energy of MCC. When the interface energy between MCC and PBAT was reduced from about 33 mJ/m^2^ to about 0 mJ/m^2^, the tensile strength of the composite increased by 5.1%, the elongation at break increased by 28.8%, the crystallization temperature increased by about 6 °C, and the storage modulus increased by 42.9%. Overall, designing the surface energy of the basic hydrophilic filler to reach a nearly-zero interface energy difference between the filler and the hydrophobic matrix is helpful to improve the interface adhesion between the filler and the matrix, and can guide the preparation of high-performance composites. This approach is a useful strategy to design green composites, and can be easily adapted to large-scale industrial production.

## Figures and Tables

**Table 1:** Contact angle and surface energy date of the samples.

Sample	CA of H_2_O/°	CA of DMF/°	*γ* (mJ/m^2^)	*γ^d^* (mJ/m^2^)	*γ^p^* (mJ/m^2^)	*x^d^*	*x^p^*
Water	——	——	72.80	29.10	43.70	0.400	0.600
N,N-Dimethylformamide	——	——	37.30	32.42	4.88	0.869	0.131
MCC	34.2	8.8	64.27	12.08	52.19	0.188	0.812
PBAT	69.5	12.1	35.54	27.67	9.87	0.779	0.211
0.2%PTFE-MCC	38.6	25.8	60.43	12.52	47.91	0.207	0.793
0.5%PTFE-MCC	69.1	29.3	35.44	22.36	13.08	0.631	0.369
1%PTFE-MCC	75.4	50.0	29.07	15.70	13.38	0.540	0.460
2%PTFE-MCC	108.0	68.5	22.48	22.33	0.15	0.9933	0.0067
4%PTFE-MCC	115.0	73.7	18.92	18.79	0.13	0.9931	0.0069
PTFE	——	——	18.6 [35]	——	——	——	——

**Table 2:** Interface energy and interaction parameter.

Composites	E1-γ_12_ (mJ/m^2^)	E2-γ_12_ (mJ/m^2^)	*φ*
PBAT/0#MCC	32.97	17.85	0.797
PBAT/0.2#MCC	28.77	15.25	0.811
PBAT/0.5#MCC	−0.94	−1.49	0.980
PBAT/1#MCC	1.83	−0.05	0.960
PBAT/2#MCC	8.00	5.88	0.918
PBAT/4#MCC	9.19	6.26	0.918

Note: the interfacial energy E1-γ_12_ and E2-γ_12_ were calculated according to the harmonic mean Equation (2) and the geometric mean Equation (3), respectively.

**Table 3:** DSC dates of PBAT/N#MCC composites.

Composites	T_g_/°C	T_hc_/°C (Melting)	T_cc_/°C (Cooling)	T_m_/°C	∆H_m_/(J/g)	χ_c_/%	∆T/°C (T_m_-T_cc_)
PBAT	−30.66	89.61	77.11	121.75	11.77	10.32	44.64
PBAT/0#MCC	−31.02	91.61	80.27	123.08	10.22	8.96	42.81
PBAT/0.2#MCC	−31.24	94.82	84.57	123.75	9.47	8.31	39.18
PBAT/0.5#MCC	−31.28	97.45	86.07	124.44	9.25	8.11	38.37
PBAT/1#MCC	−31.45	97.15	85.85	124.57	9.16	8.03	38.72
PBAT/2#MCC	−31.02	96.13	85.60	124.90	8.85	7.76	39.30
PBAT/4#MCC	−31.00	95.06	85.42	124.71	8.65	7.59	39.29

## Data Availability

The data presented are contained within the article.

## Supplementary Materials

The following supporting information can be downloaded at: https://www.mdpi.com/article/10.3390/polym14101973/s1, Figure S1: SEM images and EDS mappings referring to F element of 3h ball milled microcrystalline cellulose with different PTFE addition; Figure S2: The raw date of Figure 1; Figure S3: The raw date of Figure 6; Figure S4: SEM image of the pristine MCC particle before the ball milling; Figure S5: Mechanical properties of PBAT/0.5#MCC composites with different contents of 0.5#MCC; Figure S6: X-ray diffractograms of the PBAT/N#MCC composites; Table S1: DMA dates of PBAT/N#MCC composites; Table S2: XRD analysis of PBAT/N#MCC composites.
