# Supplementary material for: Tailoring Interfacial Adhesion between PBAT Matrix and PTFE-Modified Microcrystalline Cellulose Additive for Advanced Composites
Source: Polymers (Basel). 2022 May 12;14(10):1973. doi: 10.3390/polym14101973 (PMC9145506; doi:10.3390/polym14101973)
Supplement: Supplementary file 1 [file polymers-14-01973-s001.zip › polymers-1721330-supplementary.pdf]

Supplementary Materials: Tailoring Interfacial Adhesion Between PBAT Matrix and PTFE-modified Microcrystalline Cellulose Additive for Advanced Composites

Hongkun Wang [1,2], Xuran Liu [3], Jinfeng Liu [1], Min Wu [1,*] and Yong Huang [1,*]

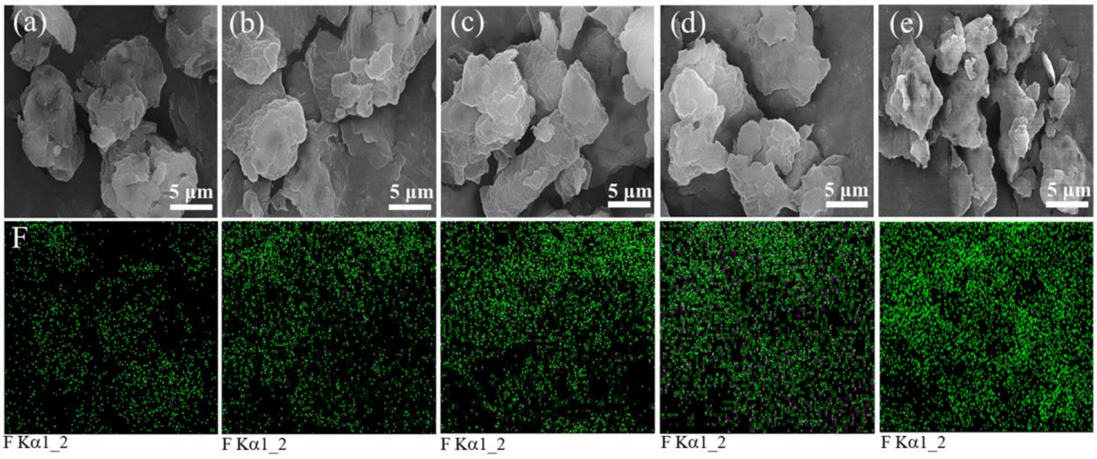

Figure S1. SEM images and EDS mappings referring to F element of 3h ball milled microcrystalline cellulose with different PTFE addition, a) 0.2%, b) 0.5%, c) 1%, d) 2%, e) 4%.

Table S1. DMA dates of PBAT/N#MCC composites

Composites	E′ at -40°C/MPa	E′ at -20°C/MPa	E″ at -40°C/MPa	E″ at -20°C/MPa	T_g/°C
PBAT	1754.5	372.1	161.8	129.5	-30.56
PBAT/0#MCC	1524.9	312.2	241.2	90.9	-36.55
PBAT/0.2#MCC	1957.7	439.9	192.3	160.6	-30.09
PBAT/0.5#MCC	2180.4	455.7	179.8	157.4	-29.73
PBAT/1#MCC	2127.9	427.9	172.5	148.5	-29.85
PBAT/2#MCC	1826.5	416.0	182.6	143.2	-29.67
PBAT/4#MCC	2086.3	535.5	181.7	183.9	-28.72

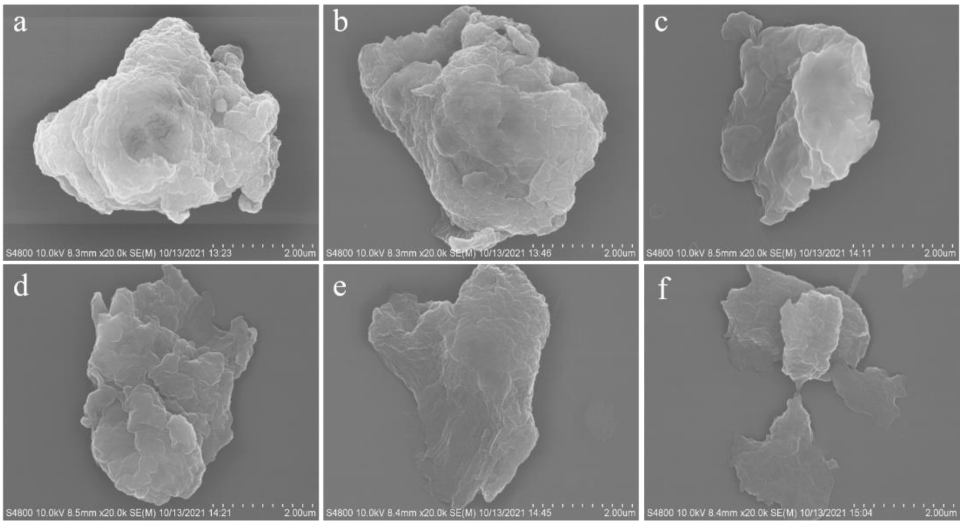

Figure S2. The raw date of Figure 1, SEM images of ball milled microcrystalline cellulose with different PTFE addition, (a) 0.2%, (b) 0.5%, (c) 1%, (d) 2%, (e) 4%.

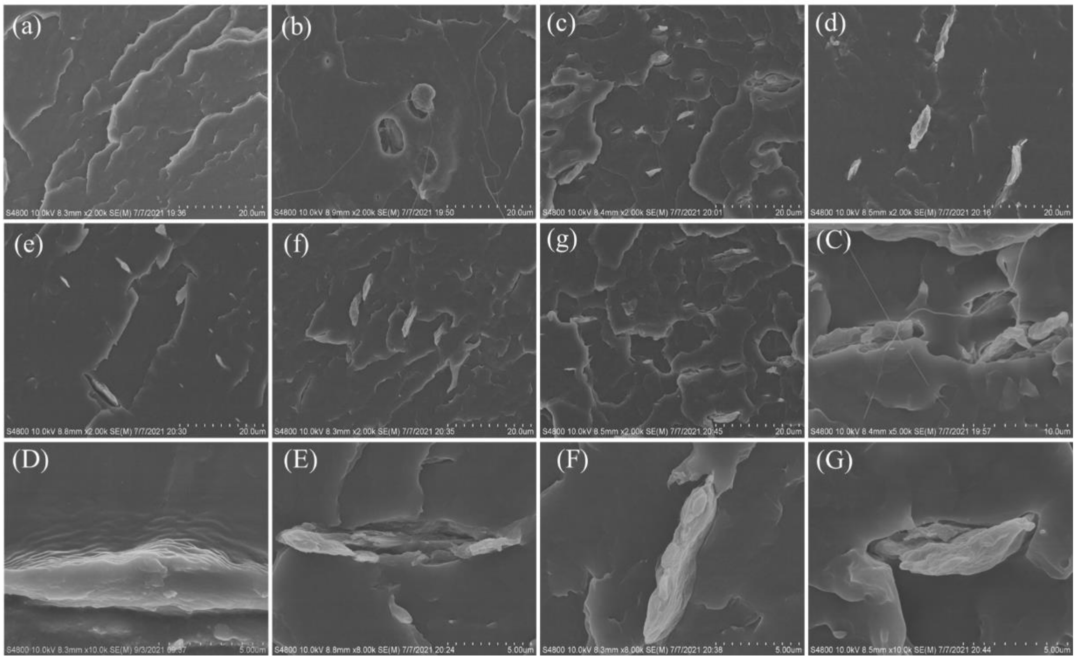

Figure S3. The raw date of Figure 6, SEM fractography of composites, (a) pure PBAT, (b) PBAT/0#MCC, (c, C) PBAT/0.2#MCC, (d, D) PBAT/0.5#MCC, (e, E) PBAT/1#MCC, (f, F) PBAT/2#MCC, (g, G) PBAT/4#MCC

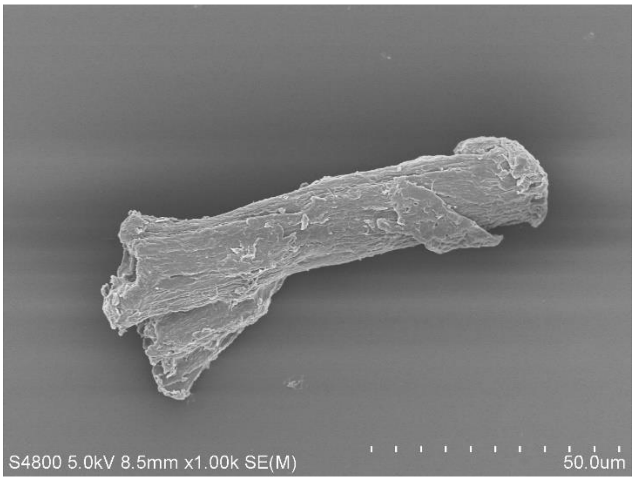

Figure S4. SEM image of the pristine MCC particle before the ball milling

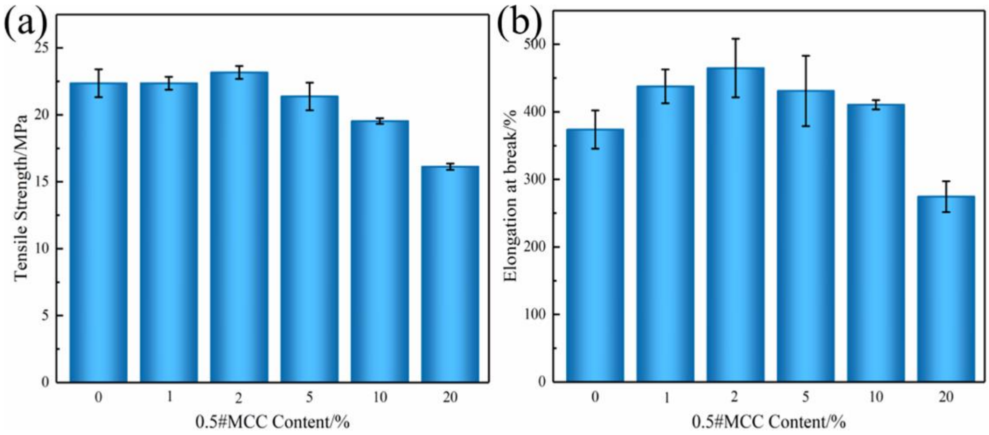

Figure S5. Mechanical properties of PBAT/0.5#MCC composites with different contents of 0.5#MCC a) tensile strength, b) elongation at break.

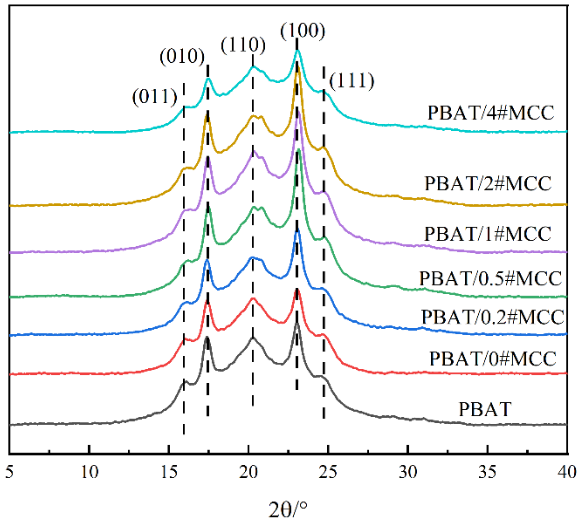

Figure S6. X-ray diffractograms of the PBAT/N#MCC composites

Table S2: XRD analysis of PBAT/N#MCC composites

**Composites**	**Apparent Crystal Size (nm)**					
	(011)	(010)	(110)	(100)	(111)	Ā
PBAT	11.78	15.47	8.92	13.52	19.86	13.91
PBAT/0#MCC	10.03	17.46	9.34	14.16	10.45	12.29
PBAT/0.2#MCC	12.15	17.50	8.44	14.92	10.69	12.74
PBAT/0.5#MCC	11.93	17.23	8.64	14.63	9.18	12.32
PBAT/1#MCC	9.96	17.27	9.32	14.24	9.46	12.05
PBAT/2#MCC	10.21	16.83	8.19	14.76	11.45	12.29
PBAT/4#MCC	11.49	16.18	9.31	13.36	12.35	12.54
